# A New Device and Method for Endothelial Graft Preparation in Descemet Membrane Endothelial Keratoplasty (DMEK): A Preliminary Feasibility and Technical Evaluation

**DOI:** 10.7759/cureus.76817

**Published:** 2025-01-02

**Authors:** Leiser Franco de Moraes Filho, Cláudia Gomide Vilela de Sousa Franco, David Leonardo Cruvinel Isaac, Ricardo Noguera Louzada, Marcos Pereira de Ávila, Leopoldo Magacho

**Affiliations:** 1 Ophthalmology, Centro de Referência em Oftalmologia, Universidade Federal de Goiás, Goiânia, BRA; 2 Ophthalmology, Universidade Federal do Rio de Janeiro, Rio de Janeiro, BRA

**Keywords:** corneal endothelium, corneal transplantation, descemet's membrane, dmek, fuchs' dystrophy

## Abstract

Background: This study compares the preparation time and the macroscopic integrity of Descemet membrane and endothelium keratoplasty (DMEK) grafts prepared using the modified submerged cornea using backgrounds away (SCUBA) technique compared to those prepared with a newly developed artificial chamber device specifically designed for DMEK preparation.

Research design and methods: This is a prospective, comparative, randomized, and experimental study. Fifty corneas from 25 donors were used. The inclusion criteria were that both eyes were of the same donor, a scleral ring greater than 3 mm, and corneas unsuitable for clinical use. Preparation time and macroscopic tissue integrity were evaluated at the end of preparation.

Results: The mean preparation time for both techniques was six minutes (p=0.2). The macroscopic integrity of the graft was similar between the inverted artificial chamber device and the modified SCUBA technique (p=1.00).

Conclusions: The upside-down artificial chamber operating with the upside-down cornea proved reproducible and macroscopically suitable for endothelial graft preparation for the DMEK technique.

## Introduction

Worldwide, around 5% of corneal diseases can lead to some form of reversible blindness [1,2]. Corneal transplantation (CT) is one of the suitable options for restoring the eyeball structure. CT is also indicated for treating corneal transparency and improving vision [3,4]. CT has provided better results for patients’ vision, mainly due to the continuous improvement of lamellar techniques, but also as a result of the advancement of surgical materials, progress in the definition of microscope images, greater efficiency in corneal preservation solutions, knowledge of endothelial function, and the development and use of more modern immunosuppressants [5,6].

Worldwide, posterior lamellar transplantation (or endothelial transplantation (ET)) has been considered the first choice among CT surgical techniques, being more indicated to treat Fuchs’ dystrophy and other diseases of the corneal endothelium [7,8]. Endothelial transplantations are advantageous because they enable faster visual recovery, maintain eyeball integrity, and cause a lower rate of rejection and refractive error in the post-operative period [6,9].

Among the techniques most commonly used in ETs today are: a) Descemet stripping endothelial keratoplasty (DSEK) [3,5,7-11] a technique in which, after being fixed in an artificial chamber, the corneal graft is dissected manually, using specific instruments in the preparation, producing a graft composed of stroma, Descemet membrane and endothelium [7,12-14]; b) Descemet stripping automated endothelial keratoplasty (DSAEK) [3,5,6-9] from which a microkeratome is used to prepare the donor tissue, which is made up of a layer of stroma, Descemet membrane and endothelium [15]; and c) Descemet membrane endothelial keratoplasty (DMEK) [3-9,14,15] a technique in which the donor tissue is made up of the Descemet membrane and endothelium complex [16].

To shorten the learning curve for graft preparation and reduce the loss ratio in the DMEK technique, this study intends to describe the development of a new device prototype of an upside-down artificial chamber (inverted artificial chamber, IAC), which allows working with the upside-down cornea, helping in the preparation of the endothelial graft for the DMEK and the DSEK technique. It aimed to compare the macroscopic integrity of the endothelial graft and the graft preparation time between the modified submerged cornea using backgrounds away (SCUBA) technique and the technique using this new prototype model [17-19].

## Materials and methods

This is a prospective, comparative, randomized, experimental study at the Centro de Referência em Oftalmologia (CEROF) of the Universidade Federal de Goiás (UFG). The Ethics Committee of the Hospital das Clínicas of the Universidade Federal de Goiás approved it on November 25, 2020 (CAAE: 40128920.8.0000.5083 - n° 4.419.698). The free and informed consent form was unnecessary as the study did not involve research directly with human beings.

The corneas used in the study were considered unsuitable for clinical use. They were collected by the CEROF/UFG eye bank from donors 40 years of age or older with positive serology for hepatitis, making CT unfeasible. The disease that led to the corneal rejection was not identified; only the positive serology was reported.

The sample was composed of corneas from both eyes of the same donor. The procedures were carried out by a single surgeon experienced in corneal endothelial transplantation techniques. Each eye of each donor was subjected to one of the techniques, and the choice between the eyes (right and left) was made randomly beforehand via the website (www.random.org). Corneas from unilateral donors were excluded.

After the initial screening, the donated corneas were examined using a slit lamp (Topcon Corporation, Tokyo, Japan), and those with lesions on the endothelium, such as tears, trauma, or lesions greater than 3 mm from the remaining sclerotic ring, were excluded. All corneas were stored correctly in solution (Optisol, Bausch Lomb, Rochester, USA). The period, in days, between the date of corneal donation and the preparation day was recorded. In both techniques described above, the cornea was positioned with the endothelium side up and stained with a 0.1% trypan blue solution (Blue Point, Oftalmopharma, São Paulo, Brazil) for 90 seconds. Five ml of BSS were used to rinse the endothelium, which was then ready to begin one of the graft preparation techniques. 

In Technique I, the donor cornea was positioned on a specific support in the modified SCUBA technique, with the endothelial side up (Barron Punch Cutting Block, Katena, New Jersey - USA). With the aid of a surgical microscope (OPMI MD, Carl Zeiss, Meditec AG, Jena, Germany) and hummingbird forceps, holding the sclerotic ring to fix the cornea, a Sinskey hook was used to dissect the endothelium Descemet membrane (EDM) 360 degrees between 0.5 and 1 mm posterior to the trabecular meshwork. The process was carefully developed to avoid penetrating the stroma.

Two ml of balanced salt solution were used to submerge the EDM complex under tension. Using a Sinskey hook and straight forceps, the EDM border was identified and carefully pulled toward the center of the cornea to find possible tears to be handled and, if necessary, with the center remaining adhered. The EDM was repositioned on the stroma and subjected to partial trephination at 8 mm, after which the tissue was detached.

**Figure 1:**
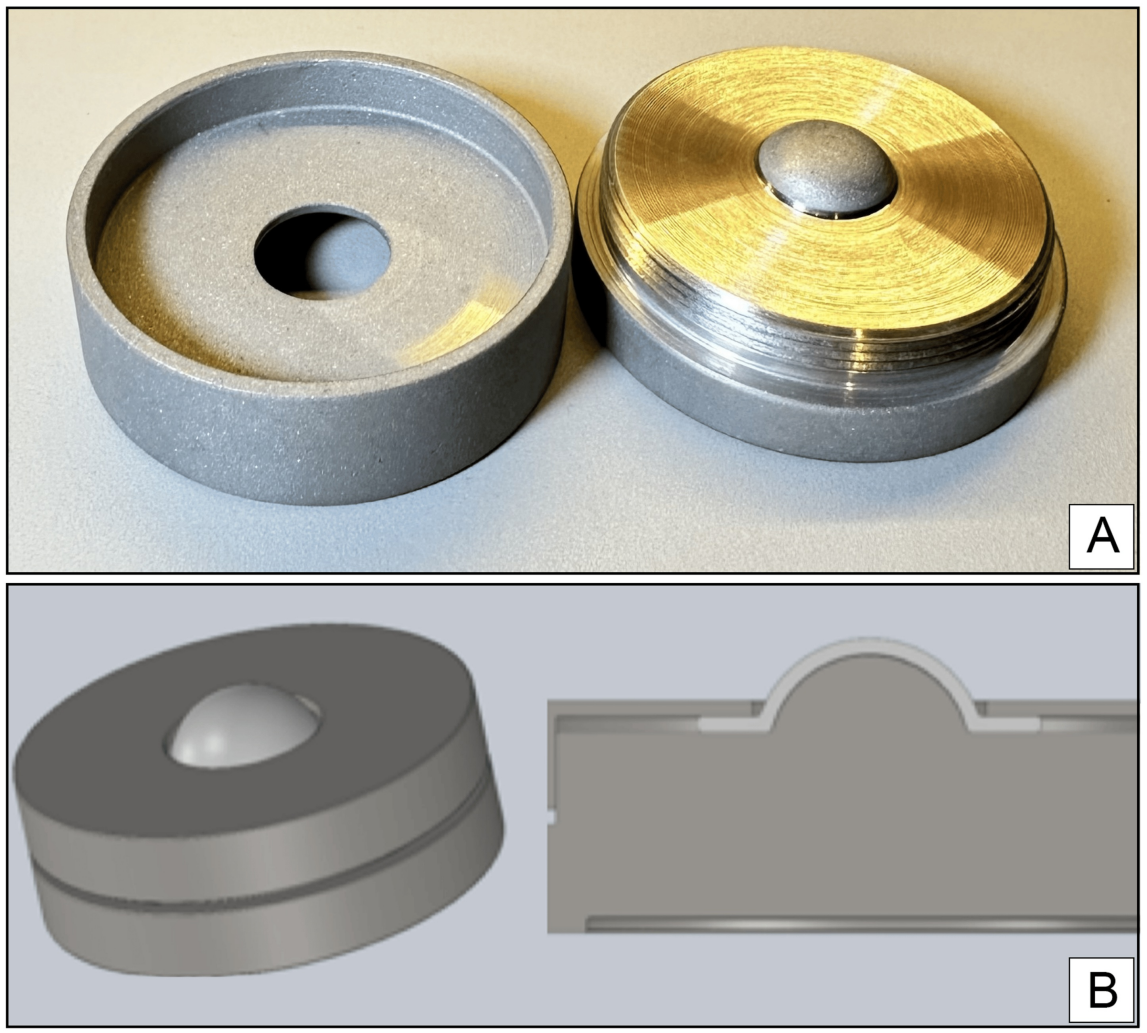
(A) Inverted artificial chamber (IAC) device. (B) The IAC device's schematic model illustrates its configuration for corneal preparation in an upside-down orientation.

Technique II used an upside-down artificial chamber device (Figure 1) with a stainless steel base 4 cm in diameter, 1.3 cm high, and 0.3 cm elevated in the center, where the corneal epithelium rests. This chamber has a thread at the lateral end, allowing the tension ring to be screwed on and attached to the sclerotic ring of the donated cornea. The tension ring attached to the base has a central hole that exposes the corneal endothelium.

**Figure 2:**
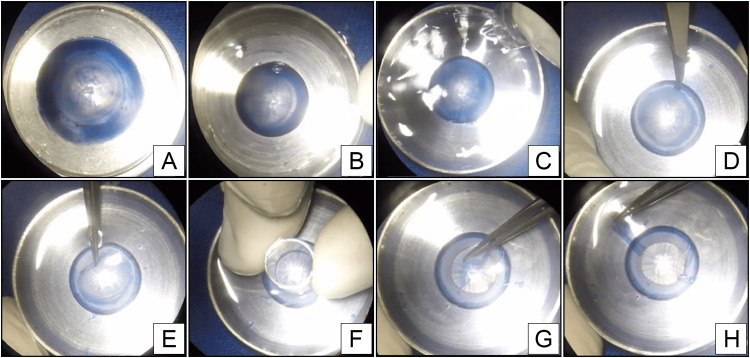
Stages of the procedure. (A) Positioning the upside-down cornea on the base. (B) Fixing the cornea with the endothelium upwards. (C) Covering with BSS. (D) Superficial peripheral dissection. (E) Peeling the endothelium. (F) Superficial trephination. (G) Finalizing the endothelium removal. (H) Finished endothelial graft.

After staining with 0.1% trypan blue for 90 seconds, the cornea was put upside-down and positioned in the IAC, with the endothelium facing upwards (Figure 2). The tensor ring securing the artificial chamber with raised edges was screwed onto its base, and the BSS was laid on the cornea. With the non-cutting side of a #11 scalpel blade (Feather Surgical Blade, Osaka, Japan) positioned between 0.5 and 1 mm from the trabecular meshwork and resting on the edge of the tensor ring (Figure 2), the artificial chamber was rotated clockwise around its axis, so that the blunt tip of the scalpel produced a 360-degree cleavage plane between the Descemet membrane and endothelium complex and the stroma.

With the aid of point forceps and a Sinskey hook, the border of the EDM complex was identified and pulled toward the center. An 8 mm trepan was used to regularize the borders and complete the dissection of the whole tissue (Figure 2).

For a better understanding, the following criteria were observed during the study:

Preparation time

An external evaluator recorded the preparation time using a digital timer. After staining the cornea for 90 seconds with 0.1% Trypan Blue, the timer was started, but it was stopped when the Descemet membrane and endothelium complex were removed entirely.

Final macroscopic tissue integrity

A third researcher, an ophthalmologist licensed by the Brazilian Council of Ophthalmology, used a surgical microscope (Zeiss OPMI MD, Carl Zeiss, Germany) at the end of the preparation to assess the suitability or unsuitability of the Descemet membrane and Endothelium complex for use in endothelial transplantation. Suitable tissues were those with preserved regularity: Up to three tears smaller than 2 mm from the edge of the tissue after 8 mm trephination. Tissues were considered unsuitable in cases with tears greater than 2 mm on the edge of the donor tissue.

Statistical analysis

A computer with Windows system and IBM SPSS Statistics for Windows, Version 21 (Released 2012; IBM Corp., Armonk, New York, United States) were used to process the data, qualitatively analyzing the variables obtained, using absolute and relative frequencies to present the differences evaluated using Fisher's exact test and the Chi-square test. The quantitative variables were processed using the Kolmogorov-Smirnov test, with normal distribution for variables p > 0.05, presenting them in their mean, standard deviation, median, minimum, and maximum variations to compare them between the IAC and SCUBA surgical techniques, using the Mann-Whitney test with non-parametric distribution, adopting 5% (p ≤ 0.05) for the significance level.

## Results

To conduct this research, we prepared the fixed and upside-down cornea using the IAC device, following the guidelines supported in the literature on manual dissection techniques [9]. Comparing both techniques, which have different principles but similar approaches to preparing the donor graft for DMEK surgery, is essential for identifying the advantageous aspects of each modality in terms of post-surgical results [17,20-24].

The corneas used were prepared for the use of one technique in each eye (modified IAC-SCUBA) in an attempt to reduce the bias of time of corneal preservation, time between death of the donor, and collection of the corneas, age of the donor, characteristics of the adhesion between the stroma and Descemet membrane-endothelium complex associated or not with the clinical characteristics of the donor.

Fifty corneas from 25 donors were assessed: 25 corneas from the right eye, 25 corneas from the left eye (same donor), 15 corneas from male donors, and 10 corneas from female donors.

The mean age of the donors was 56.5 years (+/- 11.9 years), with donors aged between 40 and 75. The average preservation time was 192.80 days (+ 120.02), with the shortest preservation time being 20 days and the longest 338 days.

Table 1 compares eye, time, and tissue viability between the IAC and modified SCUBA techniques. Each row compares data between the two groups (IAC and SCUBA), with different measures for preparation time and tissue integrity, including descriptive statistics and p-values indicating statistical significance.

**Table 1:** Comparison of techniques for all corneas in the study. (a) Chi-square test; (b) Mann-Whitney test; (c) Fisher’s exact test. IAC: Inverted Artificial Chamber; SCUBA: Submerged Cornea Using Backgrounds Away

Characteristics	IAC	SCUBA	p
Right eye N(%)	11 (44.0%)	14 (56.0%)	<0.001^a^
Left eye N(%)	14 (56.0%)	11 (44.0%)	
Preparation time (minutes)			
Mean ± standard deviation	6:02 ± 1:41	6:01 ± 1:59	0.290^b^
Median [min-max.]	5:33 [3:34 –10:03]	6:50 [3:32–08:20]	
Tissue integrity			
Loss of integrity N(%)	2 (8.0%)	1 (4.0%)	1.000^c^
Healthy N(%)	23 (92.0%)	24 (96.0%)	

One donor showed failure of macroscopic integrity in graft preparation with both techniques due to muscular adhesions between the stroma and the Descemet membrane. This finding pertains to a donor exhibiting atypical muscular adhesions between the stroma and the Descemet membrane. We suspect these adhesions could be associated with a preexisting ocular condition or degenerative changes, which hindered the membrane's separation and ultimately led to the failure of macroscopic integrity in both graft preparation methods. The use of the IAC and modified SCUBA techniques as a control was evaluated because the latter is a method considered the gold standard for graft preparation [9,17,21] and is routinely used by the evaluator.

## Discussion

The number of endothelial transplants performed in several locations worldwide has grown significantly in recent decades, increasing the demand for corneal donation [15]. There are records of an increase in the proportion of approximately two hundred thousand ETs annually performed worldwide [14]. However, these rates cannot effectively meet a substantial portion of the demand [9].

The use of the DSAEK [5-15,18,20-24] and DMEK [1-25] techniques is more frequently reported in the literature. Both techniques are suitable and beneficial for improving objective and subjective visual acuity and reducing rejection rates compared to penetrating corneal transplantation [10]. Literature needs to include comparative records to determine the best treatment option [10] precisely. In the DMEK technique, however, several studies show that visual rehabilitation is faster and restores final visual acuity compared to other techniques, with lower rejection rates for the donor graft [7,20,21].

In DMEK, the preparation of the endothelial graft is considered a critical step, given that in most countries, the surgeon performs this preparation before the procedure [5,13,17]. The literature describes different techniques for preparing the graft in DMEK, such as the SCUBA technique [9,17], the peripheral blunt dissection [14,16], the "no-touch" Melles technique [5,9,17,18], the Muraine technique [9], and pneumatic dissection techniques [14]. Each option has its uniqueness and challenges regarding the learning curve [1,3,9,18].

The first studies developed using the DMEK technique showed donor tissue loss during preparation ranging from 19% to 53% [18-20]. However, the technique was refined, leading to a significant decrease in tissue loss [12]. In a series of 40 corneal donor tissues, Maharana et al. described a success rate close to 87%, with total success for 66.6% of the tissues and partial success for 20% of the tissues in preparing the graft for DMEK surgery [20].

One of the similarities between the techniques relates to the initial cleavage point of the Descemet membrane and endothelium [5,9,4,16,21,24], which occurs between 0.5 and 1.0 mm from the trabecular meshwork. Another similarity lies in the dissection method and manual traction of the Descemet membrane, endothelium, and stroma [1,3,6-8,11-15,20,23]. The initial hypothesis was that fixing the corneal button, IAC would provide excellent stability in the graft dissection process, improving the use and learning curve when preparing the endothelial graft for DMEK. Both techniques were shown to be equivalent in maintaining the macroscopic integrity of the donor graft for DMEK surgery.

The IAC technique and the modified SCUBA technique did not present statistical difference in the preparation time of the donor graft, with a mean time of 6.02 minutes (± 1:41) for the IAC technique and of 6.01 minutes (± 1:59) for the modified SCUBA technique (p=0.29). Time assessment is an important datum, as most surgeons need to prepare the graft immediately before the surgical procedure [1,5,10,22-24], so optimizing preparation time can mean improving the performance of surgeries in the operating room [3,6,23].

The IAC technique showed a loss of macroscopic integrity of the donor tissue, a tear greater than 2 mm of the graft edge after its final 8% trephination (two cases out of 25). In comparison, the modified SCUBA technique showed a rate of 4% (1 case out of 25, p=1.0). In one donor, the cornea of both eyes presented multiple horseshoe ruptures, thus hindering proper management. This is in line with data found in the study by Tenkman et al., describing that the chance of the fellow eye presenting a horseshoe rupture was 80% in cases where these occurred in the first eye, with multiple horseshoe ruptures due to strong adhesions in Descemet membrane and stroma, hampering the endothelial tissue management [25].

Given the longer time the researcher had been using the modified SCUBA technique when preparing the grafts for DMEK, there is a potential influence bias in the final results since, ideally, the scenario should include a researcher inexperienced with both techniques or who used both techniques at similar times, to assess the learning curve better and compare the final result.

Loss of integrity only sometimes derails the clinical use of endothelial graft, as there are studies using half of the endothelial graft or even irregular grafts [3,6-8,14,15,19,22,25].

According to the literature, a longer preservation time of the corneas in their solution medium (Optisol; Bausch Lomb, Rochester, USA) at an adequate temperature may cause greater fragility to the Descemet membrane and endothelium complex [25]. Tenkman et al. recommend preserving corneas in Optisol for 14 days to preserve the functionality of the endothelium [25]. However, a longer preservation time did not influence macroscopic integrity, with tear-related macroscopic tissue loss similar to that reported by other authors [21]. 

In a study by Wasilewski et al. that analyzed rabbit corneas fixed upside-down in an IAC for 10 minutes, junctional lesions were identified in 3.58% of the endothelial cells through the impregnation of Alizarin red dissolved in isotonic solution (0.2g/100ml) and placed in contact with the endothelial surface of the membrane for two minutes and then for another minute, to highlight better and impregnate the cell junctions, indicating an injury in the stretching of the tissue when putting it upside-down [26]. The process of apoptosis in endothelial cells due to intercellular junctional lesions related to tissue manipulation during preparation still needs to be fully understood [27,28]. It should be noted that specular microscopy cannot identify junctional lesions caused by the manipulation of endothelial tissue. In the initial stages of validating a new corneal graft preparation technique for DMEK, the analysis of the endothelial graft’s macroscopic integrity and preparation time were prioritized.

The Descemet membrane and endothelium graft preparation with the IAC exhibited a similar success rate regarding macroscopic integrity for DMEK surgery compared to with the results of the modified SCUBA technique (p=1.0). Endothelial analysis was a limitation in this study, as the long preservation time of the corneas made this analysis impossible.

Significantly, the longer preservation time did not influence macroscopic integrity in this study. Since our primary objective was to evaluate the device's applicability by assessing macroscopic outcomes, these results support its feasibility despite prolonged preservation. As the technique improves and the examiners become more experienced, especially with the new IAC device prototype, better results may be found in new studies, which are necessary to verify the endothelial loss and the learning curve of the DMEK donor graft preparation technique and compare its results with techniques already established in the literature.

## Conclusions

The upside-down technique for graft preparation using the IAC device presented a similar rate of macroscopic integrity preservation for DMEK surgery compared to the modified SCUBA technique, with no statistically significant difference in donor graft preparation time. Although endothelial cell assessment was impossible due to prolonged preservation times and associated tissue fragility, the overall results suggest that the IAC technique may achieve or surpass established methods with increasing familiarity and proficiency. Future studies are needed to understand endothelial cell loss better, optimize learning curves, and compare the long-term outcomes of this technique against other standard graft preparation methods in the literature.
